# Human Papillomaviruses-Associated Cancers: An Update of Current Knowledge

**DOI:** 10.3390/v13112234

**Published:** 2021-11-06

**Authors:** Ena Pešut, Anamaria Đukić, Lucija Lulić, Josipa Skelin, Ivana Šimić, Nina Milutin Gašperov, Vjekoslav Tomaić, Ivan Sabol, Magdalena Grce

**Affiliations:** Division of Molecular Medicine, Ruđer Bošković Institute, Bijenička Cesta 54, 10000 Zagreb, Croatia; Ena.Pesut@irb.hr (E.P.); Anamaria.Djukic@irb.hr (A.Đ.); Lucija.Lulic@irb.hr (L.L.); Josipa.Skelin@irb.hr (J.S.); Ivana.Simic@irb.hr (I.Š.); nmilutin@irb.hr (N.M.G.)

**Keywords:** human papillomaviruses (HPV), oncoproteins, proteomics, epigenetics, biomarkers, cancer, therapeutics, immunology

## Abstract

Human papillomaviruses (HPVs), which are small, double-stranded, circular DNA viruses infecting human epithelial cells, are associated with various benign and malignant lesions of mucosa and skin. Intensive research on the oncogenic potential of HPVs started in the 1970s and spread across Europe, including Croatia, and worldwide. Nowadays, the causative role of a subset of oncogenic or high-risk (HR) HPV types, led by HPV-16 and HPV-18, of different anogenital and head and neck cancers is well accepted. Two major viral oncoproteins, E6 and E7, are directly involved in the development of HPV-related malignancies by targeting synergistically various cellular pathways involved in the regulation of cell cycle control, apoptosis, and cell polarity control networks as well as host immune response. This review is aimed at describing the key elements in HPV-related carcinogenesis and the advances in cancer prevention with reference to past and on-going research in Croatia.

## 1. Introduction

Although the infectious origin of bovine warts was initially described by O. Magelhaes in Brazil (1920) [1], early studies of cell-free transmission of bovine papillomaviruses were confirmed by C. Olson and R.H. Cook (1951) [2]. In the early 1970s, in Poland, S. Jablonska et al. demonstrated human papillomavirus (HPV) involvement in squamous cell carcinoma of the skin in patients with *epidermodysplasia verruciformis* [3]. Later on, the same group, in collaboration with G. Orth et al. in France [4], characterized several new viral DNAs, i.e., HPV types in those patients. In the same period, investigation of viral aetiology of genital warts was done by H. zur Hausen et al., who subsequently postulated a possible causal role of HPVs for cervical cancer (CC) [5], which was indeed later proven [6]. Nowadays, there are more than 220 identified HPV types, which are classified in five genera: alpha-, beta-, gamma-, mu-, and nu-papillomaviruses. Mucosal/cutaneous or alpha-HPVs (65 types) are isolated from genital and oral lesions while the others from cutaneous specimens, mostly beta- (54 types) and gamma-HPVs (98 types) [7]. These discoveries dramatically changed the worldwide approach to CC diagnosis, prognosis, and prevention and subsequently led to the production of the first prophylactic vaccine against a solid tumour that is caused by a viral infection in humans [8]. Although the vaccines were primarily designed to prevent CC [9], they have proven to be useful in the prevention of other HPV-associated diseases [10].

Research on HPVs in Croatia started in the late 1980s [11], continuing later in the 1990s and until now has been carried on by M. Grce and colleagues on different aspects, including investigation of alpha- and beta-HPV, role of HPV in recurrent miscarriages, HPV genome-integration status, novel variants determination, as well as epigenetic and basic studies. The first epidemiological study by M. Grce and colleagues on alpha-HPVs in Croatian women was published in 1991 [12], and the last, which is the largest to date, was in 2017 [13]. HPV DNA presence in different anatomical sites was also investigated, including its possible role in recurrent miscarriages [14]. Multiple in-house and commercial methods of HPV detection and typing were evaluated [15]. The genome integration status was also investigated in cervical samples [16]. Subsequently, a new approach was designed and implemented for the determination of HPV variants [17], which led to identification of a novel HPV-16 variant with a duplication in the E1 region and exhibiting a lower oncogenic potential [18,19]. Further epidemiological research on alpha-HPVs was also done in the Croatian male population, including both the anogenital [20] and the oral region [21]. An additional study was done on beta-HPVs in the oral region, raising interesting findings [22]. In the last decade, the focus was on the epigenetic studies in HPV-associated lesions and cancer in different sites [23,24,25]. Moreover, additional efforts were orientated to studies on basic research of HPV-induced oncogenesis at different sites [26,27].

Given the number of studies about HPVs and related diseases, tremendous knowledge has been accumulated in the last five decades. However, this review will focus only on the essential elements of HPV-related carcinogenesis as well as the advances in cancer diagnosis and treatment, which, in addition to vaccination, are the most important in cancer prevention.

## 2. HPV Epidemiology and Association to Different Cancers

The causal relationship between oncogenic HPV types and cancer was first established for CC, which is indeed the leading HPV-associated cancer in the world, with more than half a million cases per year (Table 1). Developing countries, where screening is less likely to have been successfully implemented, share a disproportionately large burden of CC cases worldwide, where approximatively 88% of the cases are found [28]. All other HPV-associated cancers in women and men are at least tenfold less abundant than CC (Table 1). Interestingly, the last four decades have seen an enormous increase in HPV-associated head and neck cancers (HNCs), especially in developed countries, which is most likely due to a switch of risk factors for these cancers [29].

The global HPV prevalence in women with normal cervical cytology was estimated to be ~10%, varying between regions but being the highest in Africa and Latin America and the lowest in North America, Europe, and Asia [30]. In all regions, HPV prevalence was the highest in women under 35 years of age, decreasing in women of older age, but with a second peak of HPV prevalence in women aged 45 years or older, especially in Africa, South and North America, and Europe [30].

In May 2018, the Director-General of the World Health Organization (WHO) called for action towards the elimination of CC as a public health problem [31]. The global strategy for the period 2020–2030 was adapted in January 2019 with three major goals: (1) HPV vaccination of 90% of girls by 15 years of age; (2) 70% of women between 35 and 45 years of age screened with a high-performance test; and (3) 90% of women diagnosed with cervical disease treated (precancerous) or appropriately managed (invasive cancer). In most developed countries in Europe and North America, all three goals are likely to be achieved, while in the rest of the world, this will be challenging. However, with the international aid to developing countries for vaccine implementation and thoughtful testing and treatment strategies, these goals should also be reached. In addition to eliminating CC, reduction of other HPV-associated cancers will likely be accomplished by this global strategy, particularly because of the widespread acceptance of HPV vaccination of both the female and male population.

**Table 1:** Global burden of HPV-associated cancers.

	Average Number of Cases ^#^			Cancer Attributable to HR HPVs *			
Anatomical Site of Cancer (ICD-10 Code) **	In Women	In Men	Total	HR HPVs	HPV-16/18	Estimated Total Number	References
Cervix (C53)	604,127	/	604,127	89.5%	70.8%	540,694	[32,33]
Vagina (C52)	17,908	/	17,908	85.3%	63.7%	15,276	[33]
Vulva (C51)	45,240	/	45,240	87.1%	72.6%	39,404	[33]
Penis (C60)	/	36,068	36,068	84.6%	70.2%	30,514	[33]
Anus (C21)	29,159	21,706	50,865	95.9%	87.0%	48,780	[33]
Oral cavity and lip (C00-06)	113,502	264,211	377,713	7.4%	68.8%	27,951	[34]
Nasopharynx (C11)	36,983	96,371	133,354	7.9%	75%	10,535	[34]
Oropharynx (C09-C10)	19,367	79,045	98,412	24.9%	83%	24,505	[34]
Hypopharynx (C12-C13)	14,000	70,254	84,254	3.9%	80	3286	[34]
Larynx (C32)	24,350	160,265	184,615	5.7%	50.8%	10,523	[34]
Total	904,636	727,920	1,548,302	/	/	751,468	/

^#^ Estimated number of new cases in 2020, worldwide, both sexes, all ages [retrieved from Cancer Today: https://gco.iarc.fr/today/home (accessed on 3 September 2021)]; * percentage of HPV-positive cases; for head and neck cancer, only HPV-16 is indicated; ** ICD-10, International Classification of Diseases 10th Revision; C09, cancers of the tonsil; C10, cancers of the oropharynx.

## 3. HPV-Induced Carcinogenesis

**Figure 1:**
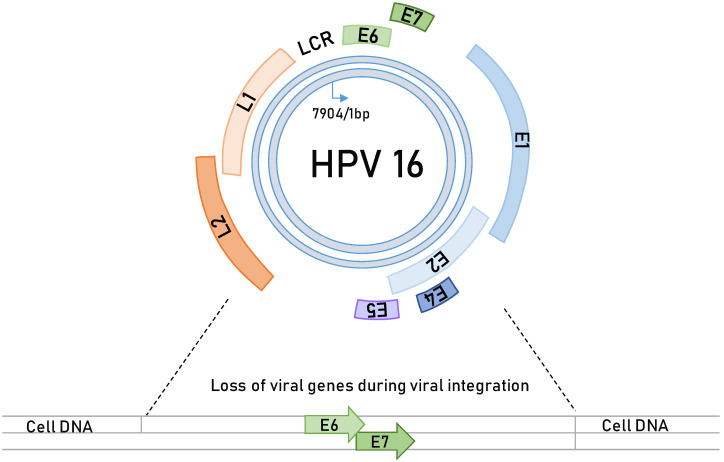
Schematic representation of HPV-16 genome. The episomal (circular) form of the viral genome comprises 7904 bp coding for a limited number of regulatory (E, early) and structural (L, late) viral proteins. LCR (long control region) is a noncoding region that regulates transcription of the viral genome. E1 codes for an ATPase (DNA-dependent adenosine triphosphatase) and DNA helicase, which serves to initiate DNA replication jointly with the E2 protein. In addition to starting replication, E2 acts as a transcriptional regulator together with the E2 response elements in the LCR. E4 serves to disrupt the keratin filaments in the upper layers of the epithelium, while E5 induces cell proliferation, angiogenesis, and apoptosis by regulating growth signalling pathways in addition to helping immune evasion. E6 oncoprotein promotes apoptosis evasion and cell-cycle deregulation. E7 promotes uncontrolled proliferation and, together with E6, induces malignant transformation. L1 and L2 genes code for viral capsid proteins, which are expressed only in the late phase of the viral life cycle. HPV DNA integration into a host cell genome is random event although it often occurs close to common fragile sites. Upon integration, most of the regulatory genes (E1, E2, E4, and E5) and the capsid genes (L1 and L2) are lost, but two main oncogenes, E6 and E7, remain uncontrollably expressed and are no longer under negative control of the E2 protein. This gives the cells integrated HPV genomes and a selective growth advantage and promotes the development of cancer.

HPVs are small, double-stranded, circular DNA viruses (genome approximately 8 kb; Figure 1), which are strictly epitheliotropic, i.e., infecting mucosal and cutaneous tissues [35]. Currently, 227 HPV types are known to persist in the human population [36], but of these HPV types, only a small number (approximately 40 alpha-types) [28] were shown to be the causative agents of more than 750,000 cases of human malignancies annually (Table 1). Of the cancer-causing HPV types, the most prevalent and investigated types are HPV-16 and HPV-18, which are responsible for more than 80% of the world’s CC burden, while the other types, frequently associated with cervical and anogenital cancers, include HPV-31, HPV-33, HPV-45, and HPV-58. Interestingly, HPV-16 appears to be the major cancer-causing HPV type that is also linked to HNC [37]. Therefore, these oncogenic viruses are classified as high-risk (HR) types [38]. Opposite to the HR types are the other HPV types, such as HPV-6 and HPV-11, which cause only benign and often self-limiting lesions and are hence referred to as low-risk (LR) types [27].

**Figure 2:**
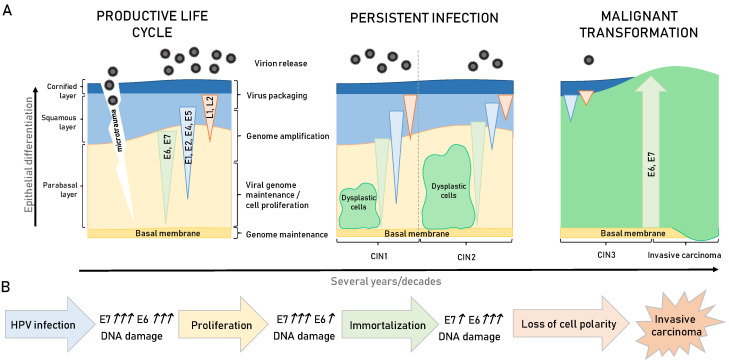
From HPV infection to malignant development. (**A**) HPV virions infect cells in the basal layer of the cervical epithelium through microtrauma. The expression of individual genes follows the differentiation of epithelial cells from the basal layer to the squamous cell layers of the epidermis with virion release from the upper layers. In the initial stage of infection, early genes are expressed, and they regulate viral replication and transcription. In persistent infection, cells in the parabasal layer become dysplastic, but the productive viral life cycle continues, with the area of cell proliferation becoming wider along with the progression of cervical intraepithelial lesions (CIN) from CIN1 to CIN2. If the host’s immune system does not resolve the viral infection, and it persists for a long period of time (several years), this can lead to pre-cancerous lesions and then to cancer development. When dysplastic cells encompass the entire thickness of the epithelia (CIN3), the productive viral life cycle is present only in the narrow area at the surface if at all. With malignant transformation, productive viral life cycle becomes abortive (green triangle—E6, E7 expression; blue triangle—E1, E2, E4, E5 expression; pink triangle—L1, L2 expression). (**B**) The expression level of viral oncoproteins E6 and E7 during HPV carcinogenesis varies as shown. The viral oncoproteins E6 and E7 mainly act on the tumour suppressors p53 and pRb, respectively. E6 binds and degrades p53 via ubiquitin-ligase E6AP, while oncoprotein E7 binds and degrades pRb via cullin-2 ubiquitin ligase complex. In the long term, infection with HR HPV types leads to uncontrolled cellular division (proliferation) without the possibility of repairing genetic defects leading to genetic instability, cell immortalization, and loss of cellular polarity, resulting finally with malignant transformation of host cells.

For HPV infection to occur, it is necessary that the viral particles (virions) use microtraumas to infect the basal cells, the primary target cells of the virus (Figure 2) [35]. After successful entry and initial burst of genome replication, the virus establishes a stable infection, during which its genomes get replicated simultaneously with cellular replication, and this is likely to occur during the wound closure [39]. Afterwards, the viral life cycle completely depends on the differentiation process of keratinocytes in the stratified epithelium. In normal epithelium, cells divide, and daughter cells migrate to the suprabasal layers, where they become contact inhibited and are likely to undergo terminal differentiation [40]. The virus does not have its own replicative machinery and therefore completely relies upon cellular enzymes. During the productive viral life cycle, two major HPV oncoproteins, E6 and E7, perform crucial activities in targeting numerous cellular processes involved in cell-cycle regulation and apoptosis, in this way creating a pseudo-S phase, which allows viral genome amplification in a highly differentiated cell population [35]. Under normal circumstances, a replicative cycle is effective and highly controlled; however, for reasons still not completely understood, this control collapses, mostly during persistent infections, and the viral DNA gets randomly integrated in the host cell genome. During these events, expression of most of the viral genes is lost, mostly E2, E4, E5, and L2, while E6 and E7 [41], become uncontrollably expressed leading to cellular transformation, immortalization, and malignant transformation (Figure 2).

The process of E6/E7 oncogene integration in the host genome results in their uncontrollable and upregulated protein expression, which is considered to be the key step in HPV-mediated carcinogenesis [42]. Cellular expression of these two viral oncoproteins was shown to be active many years after the primary immortalizing events, and it appears to be indispensable for maintaining transformed cervical tumour phenotype as well as the continual proliferation of cells isolated from these tumours. This was demonstrated in many studies using various methods to silence expression or block activities of these two oncoproteins. These approaches included peptide aptamers to target E6/E7 or antisense RNA against E6, siRNA silencing of E6/E7 expression, RNA aptamers targeting E7, and intrabody targeting of E6 [43]. In addition, ablation of E7 expression, even in tumours from the late stages of malignancy, leads to tumour cessation in transgenic animal models of HPV-induced oncogenesis [44]. Likewise, multiple studies have demonstrated the importance of the joint action of E6 and E7 for primary keratinocyte immortalization. In this setting, E6 was shown to be involved in modulation of cell survival pathways, while E7 was the main driver of cellular proliferation [45]. On the contrary, in experiments where either E6 or E7 were individually expressed, they did not exhibit any significant effect on cellular immortalization. These observations were further confirmed in transgenic mouse models for CC in which E7 upregulated proliferation and centrosome copy numbers and stimulated development of multifocal microinvasive CC. In the same experimental setting, E6 increased centrosome copy numbers and ablated cellular p53 expression; however, it did not cause neoplasia or malignant transformation. Notably, at instances when both oncoproteins were simultaneously co-expressed, the results were elevated centrosome numbers and large, highly invasive cancers [46]. The exact mechanisms by which E6 exhibits its transforming capacity are still being investigated, but E6 interplay and inactivation of cell polarity regulators appears to be one of the critical aspects [26,47,48]. Interestingly, mutual E6/E7 expressions in transgenic animal modes of head and neck squamous cell carcinomas (HNSCC) induced comparable effects. In these models, E7 was shown to be a strong inducer of predominantly benign cellular proliferation, whilst E6 seemed to be mostly involved in the later stages of malignancy [49]. These processes of HPV-induced oncogenesis are represented in Figure 2B. Hence, a better understanding of the molecular mechanisms underlying E6 and E7 respective functions is a pressing need since this could be of a significant help in developing antiviral therapies against HPV-induced malignancies.

## 4. HPV Oncoproteins

### 4.1. HPV E6 Oncoprotein

**Figure 3:**
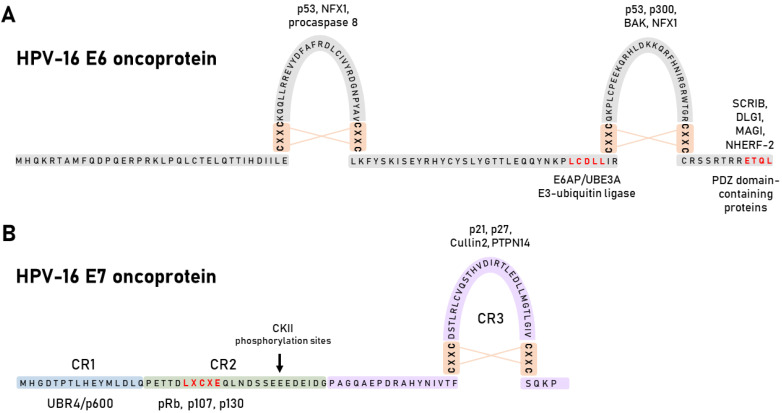
E6 and E7 oncoproteins of HPV-16. (**A**) Schematic representation of HPV-16 E6 oncoprotein amino acid sequence and motifs crucial for E6 protein integrity and activity. HPV-16 E6 contains two zinc finger domains that are involved in a subset of interactions with cellular substrates. Some of the most characterized interacting partners are depicted. (**B**) Schematic representation of HPV-16 E7 oncoprotein amino acid sequence and motifs essential for the oncoprotein stability and function. Three conserved regions of E7 are indicated (CR1-CR3) along with some of their most well-researched interacting partners as well as serines, which are subject to casin kinase II (CKII) phosphorylation, and the zinc-finger region with its interacting partners (adapted from Tomaic, 2016).

The HPV E6 oncoprotein contains 150 amino acids and has two zinc fingers created by four CXXC motifs [50] (Figure 3A). The integrity of these motifs is crucial for optimal oncoprotein function, and they are highly conserved across all HPV E6 oncoproteins [51]. The LXXLL-binding motif (LCDLL in HPV-16) is one of the conserved binding motifs of E6, with the most notable E6 substrate being an E3 ubiquitin-protein ligase E6AP/UBE3A (E6-associated protein), which it uses to induce proteasomal degradation of numerous cellular proteins [52]. The other notable domain is the PDZ (PSD95/Dlg/ZO-1)-binding motif (PBM), which is exclusively present on the C-terminus of HR HPV E6 oncoproteins and serves to accomplish interactions with cellular PDZ domain-containing proteins [53].

E6AP is the principal ubiquitin ligase and the preferred interacting partner of alpha-HPV E6 oncoproteins [54]. A stable complex between the viral oncoprotein and the ubiquitin ligase targets several cellular substrates for proteasome-mediated degradation resulting in modulations of various cellular pathways. The purpose of these modulations is to optimize the cellular environment for a productive viral life cycle, with an unintended potential to initiate the process of carcinogenesis [55]. Since E6AP was initially identified through its interaction with HR HPV E6 proteins, it was originally thought that this association was exclusive to HR HPV mucosotropic types [52], but studies showed that LR HPV-11 E6 could also complex with E6AP [56]. Furthermore, the stability and global transcriptional effects of alpha-HPV E6 oncoproteins are strictly dependent on the presence of E6AP [57,58]. There are additional ubiquitin ligases and components of the Ubiquitin Proteasome System (UPS) directed for similar activities by E6 viral oncoproteins, and these interactions with the UPS are necessary for the maintenance of the transformed phenotype [27]. HR HPV E6 interacts with the E3 ubiquitin ligases UBR5 (EDD) and HERC2 as well as with the S5a proteasome subunit, signifying the complexity of E6 association with the proteasome [59,60,61] 

**Figure 4:**
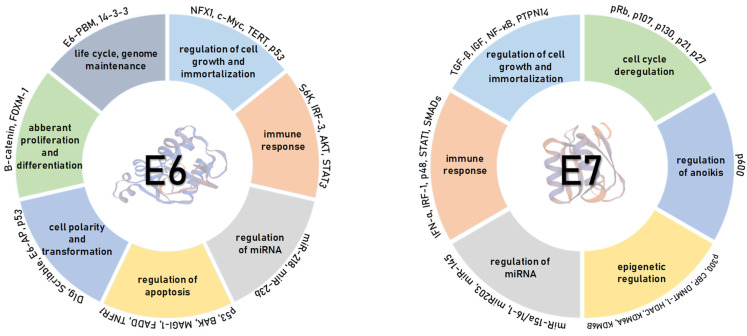
HPV E6 and E7 employ a multifaceted approach in modulating cell behaviour. The oncoproteins utilize various strategies and interact with numerous host cellular proteins to modulate various cellular processes important for productive viral life cycle and malignant progression. By the joint actions of E6 and E7, HPV can evade the host immune response and establish a persistent infection and to deregulate programmed cell death pathways, such as apoptosis and anoikis, in order to make the cells more tolerant to the changes in cell polarity and uncontrolled proliferation, which inevitably leads to cell transformation and immortalisation. Concomitantly, E6 and E7 regulate differential expression of micro RNAs in HPV-infected keratinocytes or HPV-induced malignancies. Likewise, the epigenetic signatures of various genes have been found to differentiate between lesions and normal tissue due to the interactions of oncoproteins with methyl- and acetyl-transferases.

The main oncogenic activity of HR E6 oncoproteins is the ability to interact with the p53 tumour suppressor [62]. HR E6 proteins use several mechanisms to interfere with p53 functions. Primarily, HR HPV E6 promotes p53 ubiquitination and proteasome-dependent degradation through interaction with E6AP by the formation of a trimeric complex [63]. With this interaction, E6 protects cells from cell death or growth arrest due to E7-mediated cell-cycle re-entry. E7 and E6 block checkpoint control and p53-induced apoptosis, respectively, although the remaining p53 is activated in response to DNA damage and additional cellular stressors [64]. Next, E6 proteins can also bind p53 directly and block its transcription by interfering with its DNA-binding activity [65]. Furthermore, E6 binds to the histone acetyltransferases (HAT) p300, CREB-binding protein (CBP), and histone acetyltransferase (ADA3) [66], and by these interactions, it acts as potent inhibitor of p53 transcriptional activity and contributes to the additional interference of p53 function [55]. The interference is accomplished by blocking the ability of these factors to acetylate p53 and therefore increasing its stability. As HPV infections persist for long periods, the abrogation of p53 function allows genetic mutations to accumulate in the host epithelial cell. It is important to mention that E6 also inhibits apoptotic signalling through the proteasomal degradation of pro-apoptotic Bcl2 family members BAK and BAX [67]. HR HPV E6 also upregulates the expression of anti-apoptotic factors, such as inhibitor of apoptosis protein 2 (IAP2/BIRC2) and survivin/BIRC5, and blocks the interactions of pro-apoptotic proteins, such as tumour necrosis factor receptor 1 (TNFR1), the adaptor molecule Fas-associated death domain (FADD), and procaspase 8 [55] (Figure 4).

In addition to their other functions, HR HPV E6 oncoproteins are also involved in the deregulation of the cellular DNA replication machinery. HR HPVs activate the enzyme telomerase and prevent the telomere shortening through various mechanisms, enabling continued cell proliferation [68]. HPV-16 E6 induces telomerase activity through transcriptional transactivation of the telomerase reverse transcriptase (hTERT) catalytic subunit, which is an essential step in cell immortalization, along with pRb inactivation by E7 [69]. HPV-16 E6 hTERT activation appears to be one of the crucial steps for HPV-induced malignancy [55] (Figure 4).

Cell polarity is maintained by the interplay between the conserved groups of proteins that form three different protein complexes: The Crumbs, Par, and Scribble complexes [53]. So far, 14 PDZ domain-containing substrates of E6 have been identified. These cellular proteins are involved in the regulation of cell growth, polarity, and adhesion and are a part of signal transduction pathways involved in cell differentiation, proliferation, apoptosis, migration, and intracellular trafficking [47]. Most PDZ domain-containing proteins are also characterized as tumour suppressors.

As stated above, the PBM is found exclusively on the C-terminus of HR E6 proteins [53]. Most PDZ proteins that interact with E6 are targeted for proteasome-dependent degradation or in some cases have an altered cellular localization in presence of E6 [47]. E6-mediated degradation of PDZ domain-containing proteins leads to the loss of cell polarity and induction of hyperplasia by which E6 oncoprotein plays major roles in maintaining of the transformed phenotype [53]. An important factor in E6-PDZ target recognition is the specificity, and different E6 proteins interact with different PDZ domain-containing proteins [26,53].

Interestingly, the PBM motif of HR HPV E6 oncoproteins can be post-translationally regulated via phosphorylation. Within the PBM motif is a protein kinase A (PKA) phospho-acceptor site [70], and the phosphorylation of the serine/threonine residue within the binding site inhibits PDZ domain-binding activity [71]. Phosphorylation, by either PKA or the Ser/Thr kinase AKT (protein kinase B), blocks PDZ-PBM binding and allows E6 to directly interact with the 14-3-3 zeta protein (Figure 4), a protein involved in the regulation of cellular processes (signal transduction, cell cycle regulation, and apoptosis) that are related to cancer progression [72]. Phospho-regulation of PBM and the PKA consensus recognition sequence is highly conserved between HR HPV types, which highlights the importance of the PBM and its role in HPV-induced carcinogenesis [71].

In summary, HR HPV E6 blocks apoptosis; activates the telomerase; disrupts cell polarity, epithelial differentiation, and adhesion; and influences transcription and G-protein signalling of HPV infected cells, all of which plays critical roles in the maintenance of the transformed phenotype in HPV oncogenesis.

### 4.2. HPV E7 Oncoprotein

HPV E7 oncoproteins are acidic phosphoproteins of about 100 amino acids [73]. The amino N-terminal domain of E7 is divided into three conserved regions (CR1-3) (Figure 3B). Interestingly, a small portion of the CR1 and nearly the entire CR2 are similar to the adenoviral E1A proteins and large tumour antigens (T Ag) of Simian vacuolating virus 40 (SV40). Although E7 lacks an enzymatic activity, its transforming abilities seem to be compensated by these sequences [74]. Moreover, E7 has no significant sequence similarities with cellular proteins except for the LXCXE motif in the CR2 domain. Adjacent to the LXCXE motif in the CR2 domain is a casein kinase II (CKII) consensus phosphorylation site [75]. A number of studies have shown that E7 is differently phosphorylated by CKII during cell-cycle progression, indicating the importance of CKII for an effective viral life cycle and cellular transformation [41]. The E7 oncoprotein also contains the CR3 domain, a conserved carboxyl terminal domain that forms a uniquely folded zinc-binding structure consisting of two CXXC amino acid sequences that are generally separated by 29 amino acids [76] (Figure 3B).

As previously mentioned, E7 is a highly multifunctional protein that is crucial for the productive stage of viral life cycle [77] as well as for cancer development [46]. It has many cellular interacting partners and therefore modulates a broad range of biological activities (Figure 4). E7 mainly acts as a regulator of transcription either by activating E2F-dependent transcription or by influencing the chromatin conformation. It was previously demonstrated that HPV E7 associates with the cullin-2 ubiquitin ligase complex and uses it to induce proteasome-mediated degradation of the tumour suppressor pRb [78]. This causes the disruption of pRb/E2F repressor complexes, release of the E2F transcription factor, and hence uncontrolled G1/S-phase cell-cycle progression [78,79]. Moreover, E7 regulates pRb-related pocket proteins p107 and p130, which bind and inhibit members of the E2F family, E2F4 and E2F5, respectively, in a manner similar to pRb and with this contribute to the regulation of the cell cycle [35]. Likewise, analysis of E7 interacting proteins indicated that HPV-16 E7 interacts with E2F6, a member of the E2F family of transcription factors, directly and independently of pRb since E2F6 lacks the C-terminal binding domain [80].

Another cellular tumour suppressor, the PTPN14 protein, has been reported as a target of the E7 oncoproteins. Studies have shown that E7-mediated PTPN14 degradation seems to be independent of cullin-1 or cullin-2 ubiquitin ligase complexes, most likely requiring the p600/UBR4 ubiquitin ligase [81]. PTPN14 degradation contributes to cellular immortalization and thus to HR HPV E7-mediated oncogenic activity independent of pRb degradation [82].

Even p600 has also been characterized as a cellular target of E7, which influences cellular pathways contributing to anchorage-independent growth and cellular transformation [83]. In addition, E7 stimulates cyclin E and cyclin A, genes required for S-phase of the cell cycle, and interacts with cyclin-kinase complexes leading to abrogation of cyclin-dependent kinase inhibitors, p21 and p27 [73]. Several studies have shown that E7 interacts with the SCF (Skp1–Cul1/Cdc53–F-box protein Skp2) ubiquitin ligase complex both in vitro and in vivo. The SCF complexes are ubiquitin ligases that mediate the ubiquitination of diverse cell-cycle regulators, including p27, p130, and cyclin E, to promote the entry to S-phase of the cell-cycle [84]. This mechanism of cell-cycle disruption has been widely studied in the context of HPV-mediated oncogenesis. In addition, HPV-16 E7 may also interfere with the insulin-like growth factor (IGF) signalling, again hijacking cell survival and thereby inducing cellular immortalization [85].

Another mechanism by which E7 mediates transcription is chromatin remodelling through the interaction with the TATA box-binding protein (TBP), TBP-associated factors, and members of the AP-1 transcription factor family [86]. Transcriptional activation begins with inactive or closed chromatin, which has a specific pattern of histone modifications. Later studies have shown that E7 regulates promoter activity by interacting with class I histone deacetylases (HDACs), like Mi2β, and histone acetyl transferases (HATs), including p300, pCAF, and SRC1 [87]. Aside of remodelling chromatin structure, E7 seems to also contribute to HPV-induced oncogenesis via induction of centrosomal abnormalities. This was suggested based on findings that E7 expression stimulates an increase in the number of centrosomes either directly via stimulating centrosome duplication or indirectly as a result of intruding in the p53 pathway [35].

Besides cell-cycle regulation, E7 affects many genes and their product proteins regulated through the NFκB and IFN pathways, such as IRF and STAT1, which will be discussed in a more detail further in this review. Briefly, all of these interactions allow the evasion of innate and adaptive immunity. The HPV E7 can also affect cell growth and cellular immortalization via the immunity regulator TGFβ. E7 interacts with TGFβ-regulated SMAD proteins, sequestrating them in the nucleus and inhibiting their ability to bind to DNA [88]. Although it was primarily speculated that these E7 activities contribute to HPV-induced malignancies, it was later established that they are required for maintaining keratinocyte proliferation, which is necessary for completion of the productive viral life cycle rather than for oncogenesis itself [89]. Furthermore, E7 can inhibit the TGFβ-promoter and may contribute to the inhibition of the TGFβ receptor type I; however, the precise mechanism is yet to be found [87].

It has been more than 30 years now since the first papers describing HPV E7 transforming activities were published [90]. During that time, numerous groups worldwide put significant effort in E7 research, yielding a large number of characterized cellular interacting partners [27]. However, not all biological consequences of those interactions were found or described in greater detail. Hence, E7 will probably remain one of the main focuses in investigating HPV-induced malignancies.

## 5. HPV Biomarkers in Clinical Practice

The occurrence of false-negative results in the screening for CC and relatively low sensitivity of cytology [28] has encouraged the development of better biomarkers for cancer diagnosis and prognosis. Biomarkers enable a better diagnosis and follow-up after disease treatment. There are several approved clinical biomarkers for HPV-associated diseases, while for many, the research and evaluation is ongoing (Table 2).

Detection of cancer-causing agents, such as HPV, is the first step in cancer diagnostics that have a significant positive predictive value [91]. The most common HPV test is the detection of HPV DNA of at least 13 HR HPV types (HPV-16, 18, 31, 33, 35, 39, 45, 51, 52, 56, 58, 59, and 66) either by DNA amplification with type-specific primers or by hybridization with a cocktail of probes. There are many currently available commercial assays for HPV detection and typing [92], but the most widely used in clinical practice test for the multiplex detection of alpha-HPVs is the Hybrid Capture^®^ 2 (HC2; Qiagen, Hilden, Germany) test, which was also the first FDA-approved HPV test in the early 2000s. The main applications of HPV DNA testing are triage of undetermined and low-grade cytological abnormalities (ASC-US and LSIL) and follow-up of women after treatment for high-grade cervical disease (HSIL, CIN2+) [91,93]. In addition to primary Pap screening, HPV DNA testing has been considered and assessed as a primary screening method in larger population-based studies [91,94]. Primary screening with the HPV DNA test was shown to be more effective than a Pap test in preventing CC although the test is less specific [95]. Primary HPV DNA testing, particularly with the careHPV^®^ (Qiagen, Hilden, Germany) test, a signal-amplification test for HR HPV DNA detection, is a cheap and easy-to-use test, well-suited to be used for screening in low-resource countries [96]. For HNC, the main role of establishing HPV DNA positivity, particularly HPV-16, also has prognostic value. Numerous studies have shown that HPV-positive oropharyngeal squamous cell carcinomas have favourable prognosis [97].

Testing for HPV mRNA, particularly viral oncogenes E6 and E7, in addition to or instead of HPV DNA is clinically more informative because the test is slightly less sensitive but more specific than HPV DNA testing [98]. There are several commercially available assays that detect E6/E7 transcripts (Table 2). The E6/E7 mRNA quantitative assay as a triage test in women with ASC-US or LSIL can better identify women for colposcopy referral and strengthen the patient follow-up [99]. For HPV-associated HNC, the E6/E7 mRNA test is mostly used as the gold standard for detecting active HPV-16 infections to separate those tumours from others where HPV is only a passenger and does not drive malignant transformation [34].

Several assays focus on viral or cellular protein levels in cervical specimens [100] (Table 2). Detection of E6 oncoproteins expressed by HPV-16 and -18 have shown a high positive predictive value and a high specificity for detecting CIN3+ [101]. Thus, the assay may be a useful tool in the triage of HPV-positive women, especially among HPV-vaccinated women in low-resource settings. In HNC, detection of E6 oncoproteins is also a prognostic marker of transcriptionally active HPV infections as well as HPV E6/E7 mRNA detection [102]. Another useful biomarker is the detection of the loss of expression of L1 capsid protein of all known HPV types. It has been suggested as a marker of progressive lesions [100,103].

Overexpressed p16 can serve as a surrogate biomarker for the persistent infection with HR HPV and triage of equivocal cytology findings [104]. In addition, Ki-67 marker indicates cell proliferative capacity found in HPV-infected tissues, and immunostaining is generally in line with increasing grades of cervical dysplasia [104]. At present, the most promising cellular protein biomarker assay for triage after a positive HPV test is a dual staining for p16 and Ki-67, which has better sensitivity and specificity than single staining [105]. Wentzensen et al. suggested that dual stain reduces unnecessary colposcopy referral, cervical biopsies, and treatment compared with Pap testing [105]. Clarke et al. stated that triage with p16/Ki-67 provides better long-term risk stratification than cytology over five years and that the low risk of cervical precancer in negative dual staining of p16/Ki-67 permits safe extension of follow-up intervals for three years [106]. As for HNC, in one study, p16/Ki-67 co-expression occurred only in transformed cells, which indicates cells with proliferative activity [107]. In addition, p16 positive patients with low Ki-67 in HNC showed good local control without metastasis [107].

Detection of both minichromosome maintenance complex component 2 (MCM2) and topoisomerase II alpha (TOP2A) proteins represents another promising set of biomarkers, which has been linked to the severity of cervical lesions and the presence of HR HPV [104]. Both MCM2 and TOP2A proteins have been shown to accumulate in HPV-transformed cells, and the MCM2/TOP2A dual staining (ProEx C, BD Diagnostics, Franklin Lakes, NJ, USA) is an indicator of cells with higher proliferative capacity and cervical lesions that are more likely to progress. The MCM2/TOP2A dual staining showed an increase of both sensitivity and specificity of cervical screening in comparison with cytology and HPV triage for the detection of CIN2+ in ASC-US cases and proved to be a promising marker for the confirmation of HSIL [104]. Furthermore, the MCM2/TOP2A dual staining is very useful in histology, where it can distinguish true dysplasia from reactive/reparative changes, immature squamous metaplasia, and atrophy [103]. In addition, the MCM2/TOP2A dual staining can help assess the atypical cells to better identify true pre-neoplasia in HNC as well [108].

Besides the above-mentioned clinically useful biomarkers, there are several other biomarkers in preclinical trials, including CDC6, cyclin E, MCM5, and proliferating cell nuclear antigen (PCNA; Table 2) [109]. All these markers together with p16, MCM2, and TOP2A represent a useful panel of biomarkers that allows discrimination between LSIL and HSIL with a higher risk of progression in CC [109,110]. In addition, overexpression of telomerase has been identified as a promising biomarker of cervical dysplasia and cancer. The intensity of telomerase activity is correlated with the severity of the abnormality in biopsies and in cervical scrapings [109]. Another possible marker is the p53 protein, which is degraded by HR but not by LR HPVs. As p53 plays a key role in the induction of DNA damage repair, its impairment by HR HPV infection results in the accumulation of DNA damage and can only be detected in lesions with HR HPV presence [111]. Overexpression of C*-myc* occurs more frequently in patients with advanced cancer who have significantly poorer survival compared to patients with normal C*-myc* expression [112]. Therefore, assessment of C*-myc* expression is likely to be useful for the staging of CC and prognostic prediction.

Blood-based biomarkers are an interesting option for screening due to the widespread use of various blood-based tests and their low invasiveness. HPV-16 E antibodies and circulating HPV DNA have exhibited the strongest associations with HPV-related cancers [113]. Unfortunately, clinically validated commercial tests still do not exist. Serum detection of HPV-16 E6/E7 antibodies showed strong associations with anal and oropharyngeal cancers and weaker associations with cervical, vulvovaginal, and penile cancers, indicating that the strength of the association varies depending on the anatomic site [113]. In CC patients, the levels of antibodies against the HPV E6 and E7 decrease after treatment, and this decrease appears to be associated with a better prognosis. Sustained level of antibodies specific for HPV oncoproteins might be a specific marker for recurrent disease [114]. Circulating DNA of HPV-16 and HPV-18 also showed significant associations with oropharyngeal and cervical cancers. In addition, the rate of detection of HPV DNA in CC is higher in later-stage cancers compared to carcinoma in situ, emphasizing that circulating HPV DNA appears to reflect the real-time tumour status of newly diagnosed patients with HPV-related cancers [113].

One of the main obstacles to the use of different cellular proteins as biomarkers in clinical application has been the contradictory findings of various studies. However, complementing screening program cytology with contemporary techniques involving the detection of HR HPV and biomarkers could contribute to the increase of sensitivity and objectivity. Further efforts are needed to determine those biomarkers with a consistent profile of alterations enabling their use for routine diagnostic purposes.

## 6. Immunology in HPV-Related Cancer

In order to successfully infect and maintain viral production, HPV must use several strategies to avoid detection. Firstly, HPV antigens cannot be found system-wise, which minimizes their exposure to the immune system. Secondly, genes expressed under the early promoter are expressed in low copy numbers and only in epithelial basal cells. Thirdly, unlike most viruses, HPV does not cause cell lysis, which decreases the likelihood of antigen-presenting cells (APCs) coming into contact with virions and exposing them to immune cells [115]. Finally, the most immunogenic late HPV proteins are expressed only in cornified epithelium (keratinocytes), where the immune cells are less present [116]. However, keratinocytes themselves serve an important immune function and are considered a non-specialized type of APCs. They express pathogen recognition receptors (PRRs) that detect microbial presence and initiate innate and adaptive immune signalling [117].

**Figure 5:**
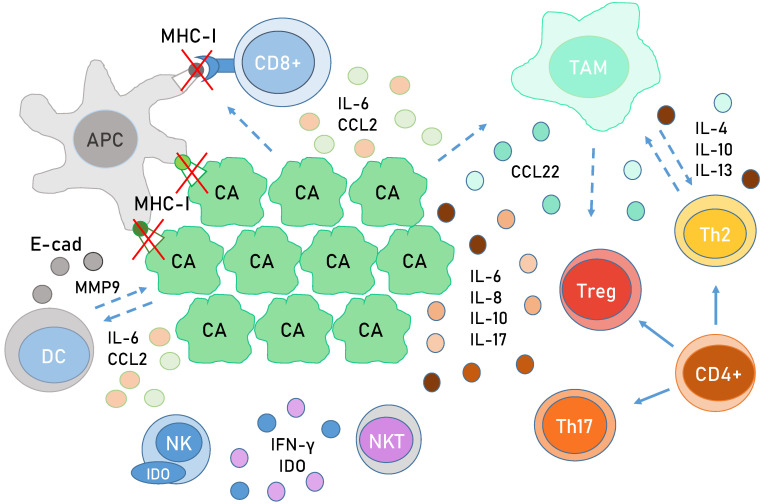
The immune microenvironment of HPV-associated cancers. HPV-infected cells express less MHC-I receptors and E-cadherin (E-cad) and, as a consequence, attract and retain less APCs and activate less CD8+ cells. Tumour cells secrete various cytokines and chemokines; IL-6 inhibits DC maturation and contributes to monocyte maturation into TAMs, as does CCL2, while immature DCs secrete the pro-tumorigenic MMP9 under the influence of IL-6. IDO secreted by tumour cells and NKT cells inhibits the cytotoxic activity of NK cells. Naive CD4+ cells mature into T helper and T regulatory cells in the tumour environment. Th2 cells secrete cytokines, which induce monocyte maturation into TAM, whereas TAMs produce the same interleukins that also promote CD4+ differentiation into Th2, creating a feedback loop. TAMs also promote the recruitment of immune-suppressive Tregs with CCL22 and the maturation of CD4+ cells into Tregs with IL-10. Th2, Tregs, and Th17 secrete IL-6, IL-8, IL-10, and IL-17, which are all pro-tumorigenic in the context of HPV-associated tumours, as they inhibit the maturation of DCs and the activation of NK cells. Abbreviations: APC, antigen-presenting cell; CA, cancer cell (HPV induced); CCL, C-C motif chemokine ligand; DC, dendritic cell; MMP9, matrix metallopeptidase 9; E-cad, E-cadherin (calcium-dependent adhesion); IDO, indoleamine 2,3-dioxygenase; IFN, interferon; IL, interleukin; NK(T), natural killer (T) cell; TAM, tumour associated macrophages; Th, helper T cell; Treg, regulatory T cell.

HPV proteins have evolved to modulate several components important for immune signalling (Figure 5). In early infections, E5 leads to a decreased expression of major histocompatibility complex (MHC) I molecules on the cell surface, which allows HPV to hide from immune cells [118,119]. The transcription of MHC I is also repressed by E7, which recruits HDACs to its promoter. HPV E7 uses a similar mechanism to repress the expression of LMP2 and TAP1, components of antigen processing machinery, effectively preventing antigen processing within the cell [116,120]. E7 also blocks endosome acidification, which is a crucial step in antigen processing and presentation [121]. In addition, E7 proteins can alter the epigenetic profile of the TLR9 promoter, decreasing the expression of TLR9, which recognizes double-stranded viral DNA [122]. On the protein level, E7 interacts with STING, the binding partner of a viral DNA detector cGAS. This interaction inhibits type I interferon (IFN-I) expression, which is important for anti-viral immune responses [123]. The IFN expression can also be initiated by IFN regulatory factors (IRF) triggered by a successful activation of PRRs [124]. HR HPV E7 employs a HDAC to block the transactivation of IRF1 [125,126], while HPV-16 E6 binds IRF3 and prevents its translocation to the nucleus, effectively blocking the expression of IFN-I [127].

All IFNs are capable of activating the JAK-STAT (Janus kinases, signal transducer and activator of transcription proteins) signalling pathway, which is considered a central communication node of the immune system, while HPV oncoproteins interfere with this pathway in multiple ways [128]. Thus, HR HPV E7 disrupts its downstream multimeric transcription factor by interacting with IRF9 and inhibiting its binding to the STAT dimer, while HR HPV E6 directly impairs STAT1 transcription [129]. This initial inhibition of signalling is thought to be important for viral replication [115]. However, increased STAT expression as well as activated JAK/STAT signalling have been found in premalignant cervical lesions and in CC [130]. STAT3 and STAT5 were found to be involved in the transcription of genes important for proliferation and survival, especially for keratinocytes in which STAT3 inhibits differentiation [131,132]. It was recently shown that STAT3 is indispensable for HPV-18 life cycle and that HPV-18 E6 is capable of inducing STAT3 activation, while HPV-31 E7 activates STAT5 [133,134]. STAT3 and STAT5 were both found to be aberrantly expressed and constitutively activated in CC, while STAT3 is also activated in HPV-positive HNC [135,136].

Like the JAK-STAT pathway, NF-κB (nuclear factor kappa-light-chain-enhancer of activated B cells) signalling has a dual role in the progression of HPV infection and cellular transformation [137]. During initial infection, NF-κB signalling is induced, which limits HPV-16 genome replication [138]. Nonetheless, it was also shown that HR HPV could suppress NF-κB signalling via the upregulation of UCHL1 [139] and by binding NF-κB coactivators in the nucleus [140]. This inhibition is considered to be important for the suppression of the innate immune response and the promotion of HPV infection. However, NF-κB signalling was found to be constitutively activated in a variety of solid tumours, including CC and HNC [141,142]. The level of expression of NF-κB components as well as the pathway activation were correlated to the progression of premalignant lesions towards cancer [143]. Considering the effects of E6 and E7, it is likely that the NF-κB pathway is activated in HPV-associated cancers by the pro-inflammatory signals coming from the tumour environment or by the change in the expression of proteins involved in NF-κB signalling.

The prolonged effects of HPV oncoproteins during a persistent infection and the changes in gene expression they cause do not only impact the infected cells themselves but also the entire microenvironment. The E7 modulation of NF-κB leads to a downregulation of CCL20, a chemokine that attracts a variety of immune cells [144]. E-cadherin is an important component in cell adhesion, and its expression is vital for the retention of infiltrating APCs. However, E6 and E7 are able to transcriptionally dampen E-cadherin’s expression by blocking its promoter activity, likely by increasing the activity of DNA methyltransferase (DNMT) [145]. Interestingly, the E-cadherin promoter is not directly methylated, but the binding of E-cadherin transactivators (Sp1 and AML-1) are greatly decreased [145]. In addition to E-cadherin downregulation, lower expression of MHC-I molecules also decreases the influx and maturation of APCs in CC and HNC, which inevitably leads to poor priming of effector T cells [146]. CC cells secrete interleukin-6 (IL-6), at least partly because of the influence of HR HPV E6 and E7 [147]. IL-6 suppresses the induction of CCR7 (C-C chemokine receptor type 7), a receptor required for the migration of dendritic cells towards the lymph node. Dendritic cells influenced by IL-6 start producing matrix metallopeptidase 9 (MMP9), which has pro-tumorigenic effects [148]. IL-6 also serves an additional purpose of stimulating STAT3 activation [149]. Taken together, all these changes create a pro-inflammatory microenvironment, which allows for tumour progression while simultaneously disabling the proper immune response.

An increased number of CD (cluster of differentiation) 8+ cytotoxic T cells was found to be infiltrating CC lesions and HNSCC, which was likely due to a high expression of pro-inflammatory chemokines and their receptors. T cells were however not activated, supposedly due to the decreased infiltration and retention of APCs and a lack of expression of viral antigens on MHC-I molecules [150]. CD4+ helper T cells are also needed for mounting a successful anti-viral response. An increase in the size of Th2 cell population along with a decrease in that of the anti-viral Th1 population has been noticed in CC, but it is unclear whether this is a direct or indirect consequence of the actions of HPV oncoproteins [151]. The T helper 17 (Th17) and the regulatory T (Treg) cells were found to be pro-tumorigenic and connected to the progression of cervical lesions to cancer [152].

Natural killer (NK) cells are components of the innate immune system capable of killing cells that have stopped expressing MHC-I molecules, which makes them very useful for the clearance of HPV-infected and cancer cells. HPVs possess a variety of methods to evade and decrease NK activity, and indeed, decreased numbers of NK cells have been found infiltrating CC and HNSCC [153,154]. HPV oncoproteins interfere with IFN production, which leads to a decreased activation of NK cells [155]. Tumour tissue, tumour associated APCs, and macrophages express immune checkpoint molecules, such as IDO, and cytokines, such as IL-10, which also supress NK cell activity [156]. Furthermore, NK cells from patients with CC express less NK-activating receptors connected to anti-tumour activity, such as NKp30, NKp44, NKp46, and NKG2D [154]. All things considered, it is clear that the modulation of NK activity by HPV oncoproteins plays an important role in the maintenance and progression of HPV-induced malignancies. Natural killer T cells (NKTs) are similar to both NK and T cells and are able to secrete various cytokines, such as tumour necrosis factor alpha (TNF-α) and IFN-γ, after activation via CD1a. NKTs are important for clearing initial infection; however, HPVs utilize proteins, such as HR HPV E5, to evade NKT cytotoxicity by downregulating CD1a [157]. Paradoxically, NKT causes immunosuppression in cervical lesions despite secretion of IFN-γ, demonstrating again the dual nature of immune modulation [158].

Tumour-associated macrophages (TAMs) are derived and differentiated from monocytes attracted to tumours, and they play an essential role in tumorigenesis. They also stimulate the differentiation of CD4+ naïve T cells into Tregs by secreting IL-10 and secrete factors, such as TGFβ and VEGF, which create conditions for tumour progression [159]. The infiltration of TAMs and their detrimental effects on tumour progression seem to be connected with HPV status, as it was found that HPV-positive HNSCC had a higher number of TAMs [160].

Even though this review provides a very condensed summary of HPV influence on the immune system, it is clear that the virus uses a myriad of tactics in order to evade immune clearance during the initial infection as well as to promote viral maintenance and propagation. During the past decade, great advances have been made in immunotherapy for cancer, but it is likely that not a single intervention with oncoprotein function could abolish the viral effects on the immune system and restore immune function.

## 7. Epigenetic Changes in HPV-Associated Cancers

### 7.1. DNA Acetylation in HPV-Associated Cancers

To enable their regulation of a diverse array of processes, the histone proteins are altered by a variety of epigenetic posttranslational modifications, including acetylation. Histone acetylation is dynamically and reversibly regulated by histone acetyltransferases (HATs) and HDACs. Histone deacetylases are critical regulators in cell growth, apoptotic programs, and differentiation. However, factors that regulate HDACs in cancers remain rather unclear [161]. Histone acetylation promotes gene expression by altering the nucleosome’s spatial structure, inducing chromatin loosening, thus increasing gene transcription and replication. Histone deacetylases remove acetyl residues and associate with condensed chromatin structures, which, in turn, suppress transcription [162]. Because of its significant role in cancer, HDACs became a potential target for cancer treatment [163].

It is demonstrated that HR HPV E7 interacts with HATs, like CBP and p300, which are involved in the regulation of many genes. E7 binding to p300 can also increase acetylation of pRb, reducing its inhibitory activity on E2F-mediated transcription. Conversely, E7 binding to HDACs can repress transcription as well as activate it [87].

As previously described, HR HPV E6 associates with not only E6AP to degrade p53 through the proteasome pathway but also with p300 to block p300-mediated p53 acetylation, which disrupts p53 stability and transcriptional activity [164,165]. Moreover, it was also demonstrated that HR HPV E6 proteins induce the rapid turnover of HAT Tip60, which is critical for activation of DNA repair and chromatin remodelling. The knockdown of Tip60 prevents p53 acetylation, which probably plays a role in the DNA damage response in HPV-positive cells. In addition, Tip60 controls the acetylation of histone H2AX, which plays an important role in DNA repair itself [166].

In the past decade, significant progress has been made in the research and development of epigenetic drugs targeting DNA methylation and demethylation, histone acetylation and deacetylation, as well as histone acetylation recognition. Many epigenetic drugs have been used in clinical trials to treat cancer and other diseases, and this field hopefully will bring a new, encouraging shift in the treatment of HPV-associated cancer [163].

### 7.2. DNA Methylation in HPV-Associated Cancer

DNA methylation is the most studied epigenetic change that influences gene expression. It is a post-replicative DNA modification that occurs mainly on the 5′-position of cytosine located in CpG dinucleotides [167]. Nowadays, it is well established that epigenetic changes play a big role in the development of CC [168]. At the moment, more than 100 genes have been confirmed to be related to the occurrence and development of CC and could be used as biomarkers to predict the occurrence of CC [169]. DNA methylation profiling can be used as a biomarker for the detection of precancerous lesions as well, particularly CIN2/3 lesions that proceed to CC [170]. However, it remains important to develop more sensitive and specific DNA methylation assays in order to improve the early detection of CC and their clinical use. In summary, specific gene promoters’ methylation, once determined, present possible good methylation biomarkers for detection of HPV-associated changes in cervix and head and neck and could be useful in clinical practice. There is also a possibility that there exists a cascade of DNA methylation processes that occurs in cervical precancerous lesions as well as in potentially precancerous lesions of HNSCC.

HPV interferes with the cellular DNA methylation machinery through E6 and E7 oncoproteins, increasing the expression and activity of DNMT1 by degrading p53 [171] or by downregulating pRb [172], respectively. In this way, some genes involved in the cell cycle (RASSF1, CCNA1, and C13ORF18) [23,171,173]—those that contribute to cancer progression by increasing proliferation, invasion, and/or metastasis (PAX1, SEPT9, CDH1, DAPK1, hTERT1, hTERT2 and HIC1) [23,171,174] and those that affect cellular differentiation (TWIST1)—are regulated [23]. Li et al. also found that, in CC containing transcriptionally active HPV-16, inactivation of tumour suppressor pathways occurs via degradation of the methylation of six key genes: MT1G, NMES1, RRAD, SFRP1, SPARC, and TFPI2; repression of viral oncogenes E6 and E7 restored tumour suppressor pathways and led to apoptosis [175]. Another study found that possible candidate methylation biomarkers, which are hypermethylated in cancer, could be transcription factors, such as LHX8, TBX20, and ZSCAN18, those involved in cell signalling (RGS7), modifying proteins (STGALNAC5), and membrane proteins (lKCNA3) [176]. The same authors found immune system effector genes significantly hypomethylated in CC tissue compared to normal tissue (AIM2, BST2, BTN3A3, and IL12RB1) as well as genes involved in viral response (AIM2, BST2, and IL12RB1), which is probably triggered by HPV oncogenes [177]. Moreover, DNA methylation might serve as a defence mechanism of the host cell to silence viral DNA [23].

Similar to CC, HPV-associated HNSCC also reveals alterations in its methylation profile [178]. Our group recently analysed the whole spectrum of well-defined clinical samples from healthy and cancer tissues, including oral lesions, with one of the most recent assays, Infinium MethylationEPIC BeadChip. We found clusters of significantly hypermethylated and hypomethylated genes in cancer tissues compared to healthy tissues. Among them, we highlighted three genes that were significantly hypermethylated in HNSCC: CDH1, SLC5A10, and TBC1D2 [25]. There are several reasons for selecting the CDH1 gene as a possible methylation biomarker for predicting the occurrence of HNSCC. Firstly, CDH1 was found to be significantly hypermethylated in cancer tissues compared to control healthy tissue as well as compared to oral lesions [25]. Secondly, CDH1 was also described by other groups as a possible methylation biomarker for the early detection and treatment of HNSCC [179,180,181]. Thirdly, methylation of this gene was already featured as a possible biomarker for other HPV-related cancers, including CC [179]. Finally, the CDH1 gene is involved in mechanisms regulating cell–cell adhesions, mobility, and proliferation, and it is recognized as a tumour suppressor gene. The genes SLC5A10 (involved in transporter activity) and TBC1D2 (involved in cadherin degradation and cell–cell adhesion) could also be possible methylation biomarkers that could help distinguish between oral potentially premalignant lesions from healthy oral tissue. Among commonly reported differentially methylated genes in HPV-driven HNSCC are genes involved in cell-cycle regulation (CDKN2A, RASSF1, and CCNA1) [182,183], cellular adhesion (CDH1, CDH8, CDH18, CDH15, CDH13, CDH19 and CDH23, ITGA4) [184,185,186], and tumour progression (TIMP3) [187]. For instance, CDKN2A gene encodes for p16INK4a and p14ARF; p16INK4a is a CDK inhibitor involved in the pRb pathway, while p14ARF is involved in the stabilization of p53 by downregulating MDM2-mediated p53 ubiquitination [178].

**Figure 6:**
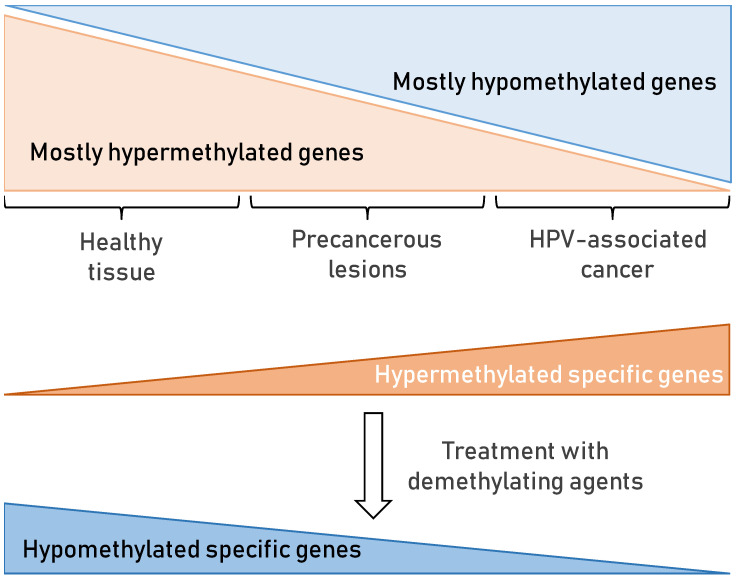
Schematic representation of DNA methylation in HPV-associated cancer. In healthy tissue, DNA methylation occurs often on most cellular gene promoters, resulting in the absence of their expression. In cancer, some relevant genes may become methylated, while most remain unmethylated. Thus, aberrant DNA methylation profile in cancer compared to normal tissue can represent a biomarker for cancer occurrence and even association to HPV infection causing that cancer. Epigenetic drugs, such as demethylating agents (DNA methyltransferase (DNMT) inhibitors), could be used to induce the expression of specific genes that would slow the progression of the cancer and enable restoration of normal phenotype. Hypermethylated genes are presented in orange and hypomethylated in blue.

Interestingly, among the possible methylation biomarker candidates in both HPV-associated cancers, CC and HNSCC, three genes were considered by different groups: CDH1, CCNA1, and RASSF1. Moreover, in line with all the above findings, since most genes have been hypermethylated in cancer, treatment with demethylating agents (DNMT inhibitors) as epigenetic therapy (Figure 6) has been shown to be beneficial in HPV-associated cancers [178,188]. Demethylating drugs should however be applied with great caution on HPV-positive patients, especially in cases where methylation of the HPV-genome has already occurred. Moreover, in such cases, demethylation of the HPV genome could possibly trigger the activity that has probably been suppressed by methylation on specific sites across the HPV genome.

In conclusion, understanding the functional role of changes in DNA methylation profiles between healthy and cancer tissue may become clinically applicable in disease diagnostics and prognostics and may guide the use of new epigenetic therapies

### 7.3. miRNA in HPV-Associated Cancer

Since their discovery in the early 1990s [189], small non-coding RNA (miRNA) molecules have been intensively studied with more than 48,000 sequences described across 270 organisms [190]. The miRNA molecules, approximately ~22nt long, are generated from longer precursors by dedicated protein machinery under tight control [191]. Their primary function is translational repression and degradation of their complementary target mRNA molecules. Thus, they serve a critical role in epigenetic regulation of various essential cellular processes from development to immunity but also play a role in many diseases and conditions [192]. Soon after it was discovered that miRNAs are conserved across different species, the field of “oncomirs”, or cancer-associated miRNA, was established [193]. It was shown that different cancer types have different miRNA signatures [194], making them promising biomarkers in cancer detection [195]. For a molecule to be a good biomarker, it should be easily accessible, specific, and sensitive [196]. Thus far, miRNA molecules appear to be both specific and sensitive, and further studies demonstrated their stability in easily accessible samples, like blood [197], urine [198], and other body fluids [199,200], as well as stability in formalin-fixed-paraffin-embedded (FFPE) tissues [201]. Despite the vast progress in the field of miRNAs as cancer biomarkers, the translation of results to a clinical setting is still challenging and usually lacks reproducibility [202]. However, research is still ongoing, and numerous miRNA molecules are investigated for their biomarker potential in many cancer types, including common and rare gynaecological [202,203], head and neck [204], gastrointestinal [205,206], and lung cancer [207]. Herein, the current knowledge regarding miRNAs as biomarkers for CC, which is the most associated with HPV, is summarized, and additional information for HNCs are provided.

In the 2000s, oncomirs were suggested to be important for CC, and at approximately the same time, their biomarker potential was discovered for other tumours [208], with first studies showing miR-21 and miR-143 deregulation in CC, while no HPV-encoded miRNAs were detected [209]. In the same year, HNSCC cell lines were profiled and deregulation of miR-21 and -205 noted [210].

Currently, there are more than 1300 publications, excluding reviews, when searching for miRNAs and CC listed in PubMed database, with more than 250 published only in 2020. To focus on the clinically most relevant information, we included keywords “biomarker” and “prognostic”, limiting the number to 395 publications (Supplementary Figure S1). Of those, 187 studies were found to be relevant and included in the final table. Within those 187 publications, 320 different miRNA molecules were highlighted as significantly deregulated in CC, of which 71 miRNA molecules (listed in Table 3) having potential clinical value with 17 miRNA molecules were highlighted by 10 or more individual publications. Among the 187 selected publications, 43 studies attempted establishing diagnostic significance of particular miRNAs, with 37 performing ROC or AUC analysis, and 26 presenting specificity and sensitivity values of particular miRNAs for detecting CC. Prognostic significance of miRNA biomarkers was described in 89 with 61 assessing survival with Kaplan–Meier analysis or 36 by Cox multivariate regression and providing survival hazard ratios.

For many of the top studied miRNAs, there is clear consistency in the direction of deregulation reported in CC, with miR-21 leading with 38 consistent studies followed by miR-20a with 14 consistent studies. On the other hand, it is difficult to find more than 4 of 187 studies reporting diagnostic accuracy for a particular miRNA (miR-20a and 205) or more than 2 reporting, albeit consistent, survival hazard ratios (miR-145, -34a, -205). Interestingly, a meta-analysis has indicated that circulating miR-205 was a good diagnostic biomarker [211].

We recently performed a similar literature review for HNC [24], where we summarized the scope of different deregulated miRNAs from 59 studies focusing on more high-throughput methods. Overall, 438 different miRNA molecules were identified as deregulated in HNC [24], which was similar to the number found in CC.

When comparing the summary of findings for CCs and HNCs, there was a noticeable overlap between the results, with the miR-21 being the dominantly studied and/or deregulated miRNA biomarker (Table 3). Not unexpectedly, miR-21 was recently indicated as a prognostic biomarker in HNC [212] and other cancers [213] by meta-analysis approaches, strongly supporting its biomarker potential, at least in some cancer types. There was also a high concordance of deregulation of the most common studied biomarkers, with the direction of deregulation being identical in both HPV-associated cancer types (last column, Table 3). A notable difference was detected for the relatively often studied miR-34a (downregulated in 9/14 studies in CC and upregulated in 6/10 studies in HNC) and for some of the less commonly studied miRNAs (miR-424, miR-192, etc.). Several of the top miRNAs were also identified as prognostic in a meta-analysis of HNC studies, including miR-21, -34a, -125b, -155, -203, -205, 210, and -218 [214].

In summary, while miRNA molecules are very heterogeneous, they are also studied very often, and with this vast amount of information on miRNAs, there are somewhat strikingly clear and repeating patterns in miRNA deregulation of HPV-associated tumours. Furthermore, many of the summarized miRNA molecules were shown to be promising diagnostic or prognostic biomarkers with either high or consistent clinically relevant indicators, like sensitivity/specificity or multivariate hazard ratios. However, it is difficult to find many studies assessing the same parameters for a particular miRNA, which precludes large-scale meta-analyses and limits the credibility needed for their routine clinical implementation. Thus, further large-scale studies or more confirmatory replication studies are needed in the future for miRNAs to be recognized as valid biomarkers despite their repeated and notable small-scale successes.

## 8. Conclusions

In the past six decades, our knowledge about the various aspects related to HPV biology has been markedly broadened. In particular, scientists investigating HPV epidemiology, oncogenesis, life cycle, viral entry, immunology, and epigenetics changes have opened new pathways for HPV vaccine design, identification of different biomarkers, and creation of a base for exploring possibilities for therapeutic intervention against HPV-induced tumours. Despite of all these valuable insights, HPV infections and associated pathologies are still a major burden globally not only in developing countries, where cervical cancer is the major public health concern, but also in economically stable regions, where HPV-mediated head and neck cancers are on the rise. Therefore, it is still a necessity to continue revealing novel HPV-associated features, which will provide a better understanding of the virus itself as well as more efficient approaches in combating HPV-mediated carcinogenesis.

## Figures and Tables

**Table 2:** HPV related biomarkers in clinical practice and research.

Methods	Indicators	Commercial Tests	Clinical Applications
HR HPV DNA detection	Present or transient HR HPV infection	Hybrid Capture^®^ 2 (HC2) HPV DNA Test (Qiagen, Hilden, Germany) Cervista HPV HR Test (Hologic, Marlborough, MA, USA) AMPLICOR HPV Test (Roche, Basel, Switzerland) careHPV™ Test (Qiagen, Hilden, Germany)	Triage of ASC-US or LSIL Follow-up women after treatment Primary screening test
HR HPV DNA genotyping	Determination of HR HPV type	INNO-LiPA^®^ HPV Genotyping (Fujirebio Diagnostics, Gothenburg, Sweden) Linear Array HPV Genotyping Test (Roche, Basel, Switzerland)	Triage of HR-HPV positive women
HR HPV E6/E7 mRNA detection	Active HPV infection	PreTect HPV-Proofer (NorChip, Klokkarstua, Norway) NucliSENS EasyQ HPV (bioMérieux, Marcy-l’Étoile, France) Aptima^®^ HPV Assay (Hologic, Marlborough, MA, USA)	Triage of LSIL
HPV proteins detection	Active HR HPV infection and progressive lesions	OncoE6™ (Arbor Vitae, Fremont, CA, USA)	Triage of HPV-positive women
	Productive HPV infection	Cytoactive^®^ HPV L1 screening set (Cytoimmun Diagnostics, Pirmasens, Germany)	Detecting CIN3
Detection of cellular proteins	Increased p16 and Ki-67 indicates cell proliferation	CINtec^®^ PLUS cytology, p16/Ki-67 dual staining (Roche, Basel, Switzerland)	Screening tool for patients younger than 30 years of age; reduces unnecessary colposcopy, cervical biopsies, and treatment
	Increased MCM2 and TOP2A indicates proliferative capacity	ProEx™ C, MCM2/TOP2a dual staining (BD Diagnostics, Franklin Lakes, NJ, USA)	Identification of cervical lesions that are more likely to progress Detection of CIN2+in ASC-US Distinguish true dysplasia from reactive/reparative changes, immature squamous metaplasia, and atrophy
	Increased CDC6, MCM5, and PCNA indicates abnormal cell proliferation beyond basal cell layers	No commercial test available, research use only	Discrimination between LSIL and HSIL Progression to cervical cancer
	Increased cyclin E indicates passage from G1 to S phase through phosphorylation of pRb and other targets		Discrimination between LSIL and HSIL Progression to cervical cancer
	Decreased p53 indicates degradation through E6 mediated ubiquitination		Presence of active HR HPV infection
	Overexpression of C*-myc*		Advanced cancer and poor survival
	Increased telomerase indicates cell immortalization		Telomerase activity is correlated with the severity of the cervical abnormalities (biopsies and smears)

Abbreviations: HR, high-risk; HPV, human papillomavirus; E6/E7, HPV early (E) expressed oncoproteins E6 and E7; ASC-US, atypical squamous cells of undetermined significance; LSIL and HSIL, low- and high-grade squamous intraepithelial lesion; CIN, cervical intraepithelial neoplasia; p16, also known as p16INK4a, cyclin-dependent kinase inhibitor 2A, CDKN2A, and multiple tumour suppressor 1; Ki-67, protein encoded by the MKI67 gene, antigen identified by monoclonal antibody Ki-67; p53, tumour suppressor protein P53; TOP2A, topoisomerase II alpha; MCM2 and MCM5, minichromosome maintenance complex component 2 or 5; PCNA, proliferating cell nuclear antigen; CDC6, cell division cycle 6; Cyclin E, limiting factor for G1 phase progression and S phase entry; C*-myc*, cellular MYC protein, transcription.

**Table 3:** Deregulation of miRNA and possible clinical applicability in HPV-associated cancers: summary from 187 and 59 selected publications for CC and HNC, respectively.

miRNA	CC								HNC		CC/HNC Similarity
	Total Studies	Up/Down Regulated	Diagnostic Utility *				Prognostic Utility **		Total Studies	Up/Down	
			N/ AUC/Sensitivity	AUC Max	Sensitivity AUC Max/Median	Specificity AUC Max/Median	N/Survival/Cox HR	Cox HR Min/Max			
miR-21	38	38/0	7/6/2	0.97	88/85	98/85	6/4/0		34	34/0	+/+
miR-145	18	2/16	3/2/2	0.85	89.1/85.0	91.7/77.4	3/2/2	1.6/2.6	15	1/13	−/−
miR-143	17	2/15	2/1/1	0.94	72.4	80	3/0/0		13	2/11	−/−
miR-125b	15	1/14	3/3/2	0.8	88/80	69/58	2/1/0		21	4/17	−/−
miR-195	14	1/13	3/2/1	0.86	88.9	78	0		11	2/9	−/−
miR-20a	14	14/0	6/4/4	0.83	77.8/72.6	97.3/82.8	3/1/0		9	7/2	+/+
miR-203a	14	3/11	1/1/1	0.65	65	62.5	2/1/0		9	3/6	−/−
miR-34a	14	5/9	4/3/2	0.95	83.3/80.6	87.5/80.1	2/2/2	0.2/0.2	10	6/4	−/+
miR-155	14	13/1	3/3/3	0.98	95/86.7	96/91.7	2/2/1	2.3	18	15/3	+/+
miR-218	13	0/13	2/2/1	0.84	67.7	60	2/2/1	0.2	8	0/8	−/−
miR-9	13	12/1	3/2/2	0.8	67/62.5	94/87	2/2/1	2.7	15	14/1	+/+
miR-100	12	1/11	1/1/0	0.88			2/1/0		20	3/17	−/−
miR-205	12	11/1	4/4/4	0.84	76.5/71	94/86.5	2/2/2	3/3.1	12	10/2	+/+
miR-200a	11	9/2	2/2/1	0.66	60	62.5	2/2/0		11	10/1	+/+
miR-141	11	9/2	1/1/1	0.94	82.8	91.7	1/1/0		8	7/1	+/+
miR-17	11	9/2	0				0		11	8/3	+/+
miR-29a	10	2/8	3/3/1	0.89	92.6	80.7	0		10	1/8	−/−
miR-210	9	6/2	2/2/2	0.97	91.6/86.3	86.8/73.8	3/2/0		12	11/1	+/+
miR-20b	8	8/0	1/1/1	0.94	83	93	0		6	5/1	+/+
miR-126	8	2/6	2/1/1	0.91	86	91	1/1/1	4	17	4/13	−/−
miR-15b	8	8/0	2/2/1	0.82	62	56	0		9	8/1	+/+
miR-224	7	7/0	1/1/1	0.89	81	93	3/2/2	4.4/6.8	11	8/3	+/+
miR-92a	7	7/0	3/3/3	0.94	94/69	87/80	1/1/0		7	7/0	+/+
miR-497	7	1/6	1/1/0	0.64			1/1/1	2	6	0/5	−/−
miR-424	7	1/6	1/1/1	0.98	100	90.9	1/1/0		11	9/2	−/+
miR-93	7	7/0	0				1/1/1	3.7	13	11/2	+/+
miR-106a	7	7/0	0				1/1/1	4	5	3/2	+/+
miR-206	6	2/4	0				2/2/1	9.1	7	2/5	−/−
miR-192	6	5/1	3/3/2	0.95	91.7/83.4	100/97	0/1/0		2	1/1	+/0
miR-486	5	4/1	3/3/0	0.9			0		12	4/7	+/−
miR-34b	5	2/3	1/1/1	1	100	100/	1/1/0		8	8/0	−/+
miR-127	5	3/2	1/1/1	0.82	75	83	0		8	1/7	+/−
miR-136	5	1/4	1/1/1	0.81	70.8	90.9	0/1/0		5	3/2	−/+
miR-142	5	4/1	1/0/0				1/1/1	3.2	12	9/3	+/+
miR-204	5	0/5	1/1/1	0.88	80.3	90.9	0/1/0		9	1/8	−/−
miR-1246	5	4/1	2/1/1	0.88	86	75	1/0/0		2	2/0	+/+
miR-335	4	1/3	0				1/1/1	0.3	3	3/0	−/+
miR-425	4	4/0	0				1/1/1	2.4	5	4/1	+/+
miR-363	4	4/0	0				1/1/1	0.1	8	6/2	+/+
miR-455	4	0/4	1/1/1	0.84	78	81.3	1/1/0		13	12/1	−/+
miR-181a	4	2/2	0				1/1/1	2.4	11	6/5	0/+
miR-26b	4	3/1	2/0/0				1/1/1	2.6	14	2/12	+/−
miR-101	4	2/2	0				1/1/1	2.8	14	6/7	0/−
miR-196a	4	4/0	1/0/0				1/1/1	3.5	14	11/3	+/+
miR-215	3	2/1	0				1/1/1	2	4	3/1	+/+
miR-638	3	0/3	1/1/1	0.73	85	46	2/1/1	2.9	0		
let-7d	3	2/1	1/1/0	0.82			0		7	3/4	+/−
miR-144	3	2/1	1/1/1	0.95	89	93	1/0/0		4	3/1	+/+
miR-494	3	1/2	1/1/1	0.91	91.7	90.9	1/1/0		4	1/3	−/−
miR-194	3	3/0	2/2/1	0.94	95.8	81.8	0/1/0		3	1/2	+/−
miR-181b	3	1/2	0				1/1/1	2.4	9	5/4	−/+
miR-370	3	1/2	1/1/0	0.82			1/0/0		5	1/4	−/−
miR-152	3	2/1	1/1/0	0.93			0		2	2/0	+/+
miR-411	3	0/3	0				2/1/1	0.4	5	0/5	−/−
miR-135a	2	2/0	1/1/1	0.83	70.8	91.8	0/1/0		3	1/2	+/−
miR-329	2	1/1	0				1/1/1	2.8	0		
miR-664	2	1/1	0				1/1/1	4.2	1	0/1	0/−
miR-299	2	0/2	1/1/1	0.98	91.6	90.9	0/1/0		5	0/5	−/−
miR-22	2	1/1	0				1/1/1	1.8	3	2/1	0/+
miR-362	2	0/2	0				2/2/2	0.4/0.5	1	0/1	−/−
miR-3162	1	1/0	1/1/1	0.87	79	50	0		0		
miR-449a	1	0/1	0				1/1/1	2.3	0		
miR-4484	1	1/0	1/1/1	0.81	72	75	0		0		
miR-2392	1	1/0	1/1/1	0.94	59	85	0		0		
miR-1254	1	0/1	0				1/1/1	2.9	0		
miR-766	1	1/0	1/1/1	0.85	79.1	82.1	1/0/0		3	3/0	+/+
miR-503	1	0/1	0				1/1/1	2.8	9	7/2	−/+
miR-451a	1	1/0	1/1/1	0.96	91	95	0		10	2/7	+/−
miR-1297	1	0/1	0				1/1/1	3.8	0		
miR-153	1	0/1	0				1/1/1	2.1	0		
miR-994	1	1/0	0				1/1/1	4	0		

* Diagnostic utility was assessed by N studies, of which some provided Area Under the Curve (AUC) analysis, and some presented sensitivity/specificity data (Sensitivity); AUC Max, maximally reported AUC value; AUC Median, median of reported AUC values; ** prognostic utility was reported by N studies, of which some provided Kaplan–Meier survival analysis (Survival) and some provided Cox multiple regression hazard ratios (Cox HR); Column Cox HR Min/Max lists minimum and maximum reported Cox hazard ratio since only two studies were found for each miRNA; CC/HNC similarity column shows the direction of deregulation, upregulated (+) or downregulated (−) in cervical cancer (CC) and head and neck cancer (HNC).

## Supplementary Materials

The following are available online at https://www.mdpi.com/article/10.3390/v13112234/s1, Figure S1: PRISMA flow diagram for selection of studies on miRNA in cervical cancer (according to [1]).

## Abbreviations

CC	cervical cancer
E	early
HNC	head and neck cancer
HNSCC	head and neck squamous cell carcinoma
HPV	human papillomavirus
HR	high-risk
LR	low-risk
SCC	squamous cell carcinoma
L	late
LCR	long control region
