# Supplementary material for: Human Papillomaviruses-Associated Cancers: An Update of Current Knowledge
Source: Viruses. 2021 Nov 6;13(11):2234. doi: 10.3390/v13112234 (PMC8623401; doi:10.3390/v13112234)
Supplement: Supplementary file 1 [file viruses-13-02234-s001.zip › viruses-1404240-supplementary.pdf]

Supplementary Figure S1. PRISMA flow diagram for selection of studies on miRNA in cervical cancer (according to [1]).

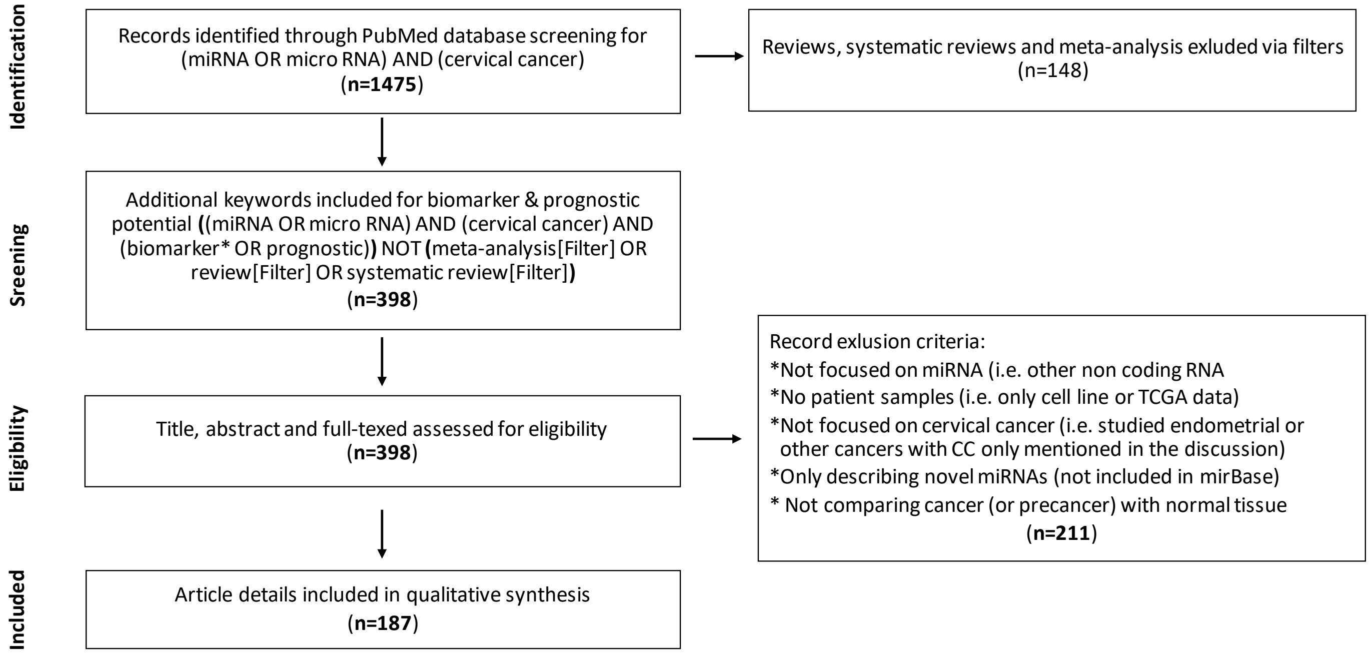

Reference

1. Page MJ, Moher D, Bossuyt PM, Boutron I, Hoffmann TC, Mulrow CD, et al. PRISMA 2020 explanation and elaboration: updated guidance and exemplars for reporting systematic reviews. BMJ. 2021 Mar 29;372:n160.
